# Calcitonin measurement in fine-needle aspirate washout fluid by electrochemiluminescence immunoassay for thyroid tumors

**DOI:** 10.1186/s13044-018-0059-4

**Published:** 2018-10-30

**Authors:** Minoru Kihara, Mitsuyoshi Hirokawa, Takumi Kudo, Toshitetsu Hayashi, Masatoshi Yamamoto, Hiroo Masuoka, Takuya Higashiyama, Mitsuhiro Fukushima, Yasuhiro Ito, Akihiro Miya, Akira Miyauchi

**Affiliations:** 1 Departments of Surgery, Kuma Hospital, 8-2-35 Shimoyamate-dori, Chuo-ku, Kobe, Hyogo 650-0011 Japan; 2 Departments of Diagnostic Pathology, Kuma Hospital, 8-2-35 Shimoyamate-dori, Chuo-ku, Kobe, Hyogo 650-0011 Japan; 3 Departments of Internal Medicine, Kuma Hospital, 8-2-35 Shimoyamate-dori, Chuo-ku, Kobe, Hyogo 650-0011 Japan

**Keywords:** Calcitonin, Fine-needle aspiration washout fluid, Medullary thyroid carcinoma, Electrochemiluminescence immunoassay, ECLIA

## Abstract

**Purpose:**

For the differential diagnosis of medullary thyroid carcinoma (MTC) on thyroid nodules, ultrasound-guided fine-needle aspiration cytology is a useful and safe procedure, but its diagnostic accuracy is not high enough. As an ancillary method to accurately diagnose MTC, the calcitonin in fine-needle aspirate washout fluid (FNA-Ct) is used. However, no data are available about cut-off values of FNA-Ct using the currently available electrochemiluminescence immunoassay (ECLIA).

**Methods:**

We investigated 180 thyroid nodules in 141 patients. After smearing, the syringe and needle used for the FNA were rinsed with normal saline (0.5 mL). The calcitonin in the washout was measured by ECLIA.

**Results:**

The FNA-Ct in the non-MTC nodules of MTC patients, non-MTC nodules of non-MTC patients, and MTC nodules were 10.6–2100 pg/mL (median 24.6 pg/mL), < 0.5–21.0 pg/mL (median < 0.5 pg/mL), and 94.9–4,070,000 pg/mL (median 177,000 pg/mL), respectively. A receiver operating characteristic analysis of the MTC nodules and the non-MTC nodules of the non-MTC patients indicated that the cut-off value was 21.0 pg/mL, leading to 100% sensitivity and 100% specificity.

**Conclusions:**

This is the first study to determine the cut-off value of FNA-Ct with an ECLIA, and we propose that the optimal cut-off value is 21.0 pg/mL.

## Introduction

Medullary thyroid carcinoma (MTC) is a quite rare malignant tumor that originates from C cells (calcitonin-producing cells), which represent < 1% of the total cells within the thyroid gland. MTC presents in a sporadic or hereditary variant [1, 2]. Serum calcitonin is the most sensitive biochemical marker for MTC for both the primary diagnosis and follow-up [3–9]. For the preoperative diagnosis of thyroid nodules, ultrasound-guided fine-needle aspiration cytology (FNAC) is a useful and safe procedure, but the diagnostic accuracy of this method for MTC is not as high as it is for papillary thyroid carcinoma (PTC) [10–15]. In their 2015 meta-analysis of 15 studies, Trimboli et al. [16] stated that the accuracy of FNAC in diagnosing MTC in patients with MTC nodules was < 50%. However, other studies have indicated that the measurement of calcitonin in the fine-needle aspirate washout fluid from thyroid nodules may significantly improve the sensitivity of the diagnosis of MTC [17–20].

Calcitonin has generally been determined using a solid-phase, two-site chemiluminescent immunometric assay or the solid two-site immunoradiometric assay that is available worldwide. In Japan, until March 2015, calcitonin was measured using the solid two-site immunoradiometric assay only. In April 2015, the measurement method in Japan was changed to an electrochemiluminescence immunoassay (ECLIA). To the best of our knowledge, no data are available about the appropriate cut-off value of calcitonin in fine-needle aspirate washout fluid (FNA-Ct) using this current calcitonin assay system in MTC patients and non-MTC patients. We conducted the present study to assess the feasibility of cut-off values of FNA-Ct obtained with the use of an ECLIA in both MTC and non-MTC patients.

## Patients and methods

### Patients

At our hospital, 103 patients had 142 thyroid nodules which were suspicious for MTC or could not be ruled out as MTC clinically and preoperatively in the period from April 2015 to November 2017. Another 38 patients had 38 thyroid nodules which were suspicious for tumors other than MTC on ultrasonography in November 2017. These 38 patients were enrolled as the control group in this study, which was approved by the Ethics Committee at our hospital. Written informed consent for their case to be used was obtained from each patient. Ultimately, a total of 141 patients were enrolled: 45 males and 96 females, median age 60 years (range 16–88 years).

## Methods

FNAC was performed by using a 22-gauge needle under ultrasound guidance. After smearing, the remaining aspirate in the syringe and needle used for the aspiration was rinsed with 0.5 mL of normal saline. The aspirate was then subjected to calcitonin measurements. The serum calcitonin was also measured in the patients suspected of having MTC. Calcitonin was measured by a laboratory (SRL Co., Tokyo) using the Elecsys® Calcitonin test system (Roche Diagnostics, Tokyo), which is an ECLIA. According to the manufacturer, the normal ranges of serum calcitonin are ≤9.52 pg/mL for males and ≤ 6.40 pg/mL for females, with a lower detection limit of quantification of 0.5 pg/mL.

All 141 patients underwent a thyroidectomy. The diagnosis of MTC necessarily involved histological and immunohistochemical examinations using antibodies against calcitonin and carcinoembryonic antigen (CEA). None of the patients had hyperparathyroidism, hepatic cirrhosis, or renal insufficiency. None of the patients had been treated with drugs that increase calcitonin secretion (such as omeprazole, beta-blockers and glucocorticoid secretagogues), and none were cigarette smokers. In all of the patients, the preoperative thyroid function was euthyroidism.

### Statistical analysis

For the comparison of continuous variables, the Mann-Whitney U-test was used. A *p*-value < 0.05 was regarded as significant. All analyses were performed using StatFlex 6.0 software (Artech, Osaka, Japan).

## Results

FNAC, FNA-Ct, and surgery were performed in 180 nodules in the 141 patients, and 39 of these nodules were diagnosed as MTCs histopathologically. The other histopathological diagnoses included papillary carcinoma (14 nodules), poorly differentiated carcinoma (two nodules), anaplastic carcinoma (one nodule), nodular goiter (93 nodules), follicular neoplasm (17 nodules), chronic thyroiditis (eight nodules), cyst (five nodules), and intrathyroid thymic carcinoma (one nodule).

The serum calcitonin values of the non-MTC patients were within the normal limits (maximum 6.7 pg/mL), whereas those of the MTC patients ranged from 86.0 pg/mL to 4970 pg/mL (median 379 pg/mL).

**Fig. 1:**
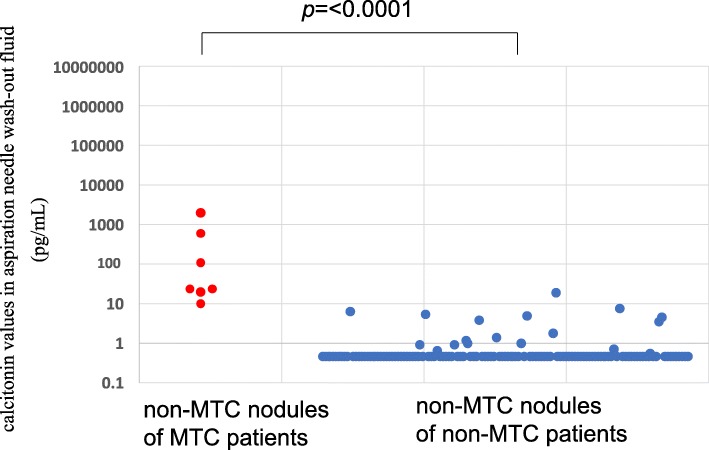
Calcitonin values in fine-needle aspirate washout fluid using ECLIA in non-MTC nodules

The FNA-Ct values in the non-MTC nodules of the MTC patients ranged from 10.6 pg/mL to 2100 pg/mL (median 24.6 pg/mL), and those values in the non-MTC nodules of the non-MTC patients ranged from < 0.5 pg/mL to 21.0 pg/mL (median < 0.5 pg/mL) (Fig. 1). These values were significantly higher in the non-MTC nodules of the MTC patients than in the non-MTC nodules of the non-MTC patients (*p* < 0.0001) (Fig. 1).

**Fig. 2:**
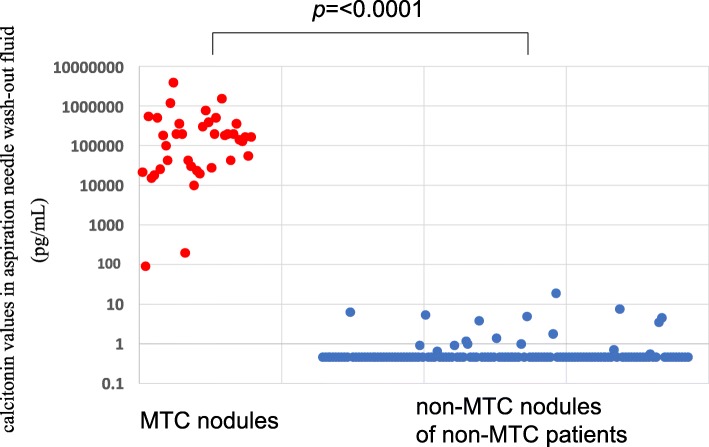
Calcitonin values in fine-needle aspirate washout fluid using ECLIA in the MTC nodules and in the non-MTC nodules of non-MTC patients

The FNA-Ct values in the MTC nodules ranged 94.9 pg/mL to 4,070,000 pg/mL (median 177,000 pg/mL) (Fig. 2), which is significantly higher than in the non-MTC nodules of non-MTC patients (*p* < 0.0001) (Fig. 2).

**Fig. 3:**
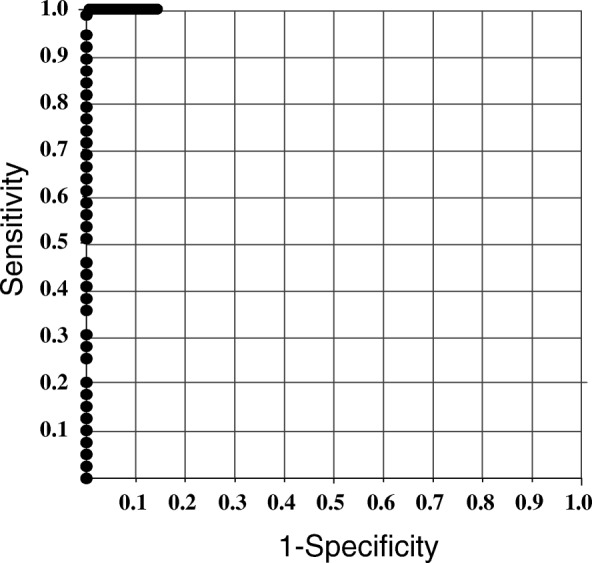
ROC curve of Calcitonin values in fine-needle aspirate washout fluid using ECLIA in the MTC nodules and in the non-MTC nodules of non-MTC patients

In a receiver operating characteristic (ROC) analysis for the MTC nodules and the non-MTC nodules of the non-MTC patients, the area under the curve (AUC) was 100%: on the basis of this curve, the cut-off value of FNA-Ct was 21.0 pg/mL, leading to 100% sensitivity and 100% specificity (Fig. 3).

**Fig. 4:**
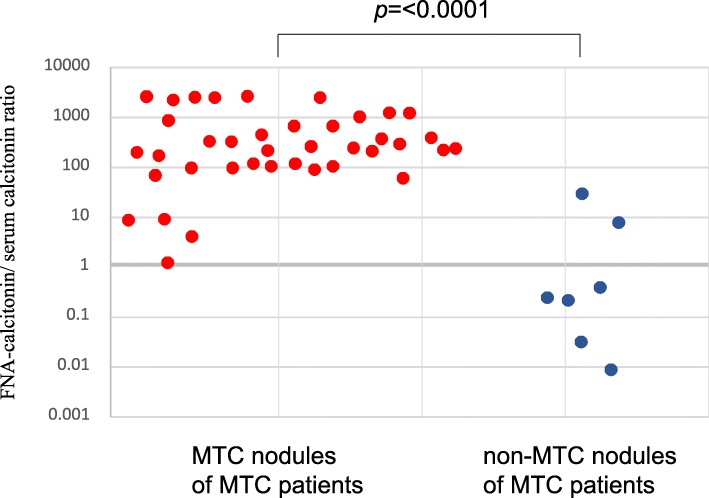
The FNA-Ct / serum calcitonin ratios in the MTC nodules and the non-MTC nodules of the MTC patients

**Fig. 5:**
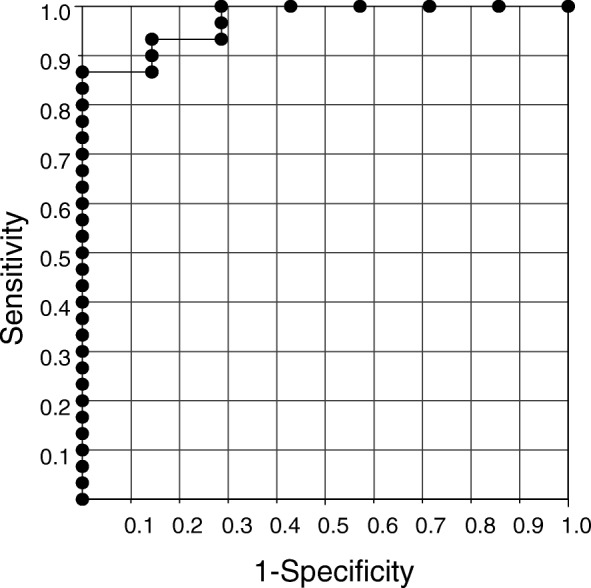
ROC analysis for the MTC nodules and the non-MTC nodules of the MTC patients

The FNA-Ct/serum calcitonin ratios in the MTC nodules of the MTC patients ranged from 0.48 to 2583 pg/mL (median 212.0 pg/mL), and those ratios in the non-MTC nodules of the MTC patients ranged from 0.009 to 39.0 pg/mL (median 0.25) (Fig. 4). These ratios were significantly higher in the MTC nodules of the MTC patients than in the non-MTC nodules of the MTC patients (*p* < 0.0001) (Fig. 4). In a ROC analysis for the MTC nodules and the non-MTC nodules of the MTC patients, the AUC was 97.1%: on the basis of this curve, the cut-off value of FNA-Ct/serum calcitonin ratio was 11.0, leading to 100% sensitivity and 86.7% specificity (Fig. 5).

The Table 1 provides the data of the non-MTC nodules of the MTC patients. The FNA-Ct value and FNA-Ct/serum calcitonin ratio were higher in the hereditary MTC patients compared to the sporadic MTC patients.

**Table 1:** Comparison of the non-MTC nodules in hereditary MTC patients and in sporadic MTC patients

Patient	FNA-Ct (pg/mL)	Serum calcitonin (pg/mL)	FNA-Ct /Serum calcitonin ratio	Sporadic or Hereditary MTC patients
1	10.6	26.8	0.40	sporadic
2	21.2	640	0.03	sporadic
3	24.4	112	0.22	sporadic
4	24.6	2600	0.01	sporadic
5	112	458	0.25	sporadic
6	623	15.9	39.2	hereditary
7	2100	282	7.45	hereditary

## Discussion

FNAC is a safe, accurate, and cost-effective method for the initial screening of thyroid nodules [21], but the diagnostic accuracy of this method for MTC is not as high as it is for PTC [10–15]. The diagnostic accuracy provided by FNAC for MTCs ranges from 50.0 to 82.4% [10, 12, 15, 16, 22], because cytologic examination results have revealed diverse appearances include a variety of cellular morphologies, non-typical cell shapes, and low cellularity in MTC [10, 11]. Serum calcitonin is the most sensitive tumor marker for MTC, and its measurement is used to follow patients with residual and metastatic MTC [23–25]. Elisei et al. [26] reported that serum calcitonin had higher sensitivity compared to cytology. However, high levels of serum calcitonin do not reveal which of multiple lesions is MTC.

A preoperative diagnosis of MTC is very important to the determination of the need for genetic testing for a germline *RET* mutation analysis and the surgical procedure. Several reports including our previous paper demonstrated that calcitonin measurements in aspiration needle wash-out fluid have increased the accuracy of diagnosis as an ancillary method [17–20]. The first papers were published by Boi et al. [17] and Kudo et al. [18] in 2007. According to the most updated American Thyroid Association (ATA) guidelines, FNA-Ct should be used to accurately diagnose MTC and avoid false-negative or inconclusive/nondiagnostic results from cytology [1]. In their review, Trimboli et al. [20] stated that different calcitonin cut-off levels (7.4–67 pg/mL) had been obtained by different criteria calculation and different assay methods. The methods used to measure calcitonin have changed worldwide over the years, from the solid two-site immunoradiometric assay (RIA) to the use of a solid-phase, enzyme-labeled, two-site chemiluminescent immunometric assay with increasing sensitivity [27]. We reported that the cut-off level to be used with an RIA was 67.0 pg/mL [18].

As noted in the Introduction, calcitonin measurement in Japan has been conducted using only ECLIAs since April 2015. We have found no data regarding the cut-off values of FNA-Ct to be used with the currently available calcitonin assay system, and we used this assay in the present study. This study is the first report of the cut-off value to be used with ECLIAs. Our ROC curve analysis for MTC nodules and non-MTC nodules of non-MTC patients revealed that the optimal cut-off value was 21.0 pg/mL, leading to 100% sensitivity and 100% specificity. On the other hand, Our ROC curve analysis for MTC nodules and non-MTC nodules of MTC patients revealed that the cut-off value of FNA-Ct/serum calcitonin ratio was 11.0, leading to 100% sensitivity and 86.7% specificity.

It is remarkable that the FNA-Ct values of all MTC nodules were high (> 94.9 pg/mL) and those of almost all of the non-MTC nodules were < 0.5 pg/mL, whereas the values of some non-MTC nodules from MTC patients were high. The FNA-Ct is directly influenced by serum calcitonin due to peripheral blood contamination [17]. The FNA-Ct values of six of the seven non-MTC nodules in MTC patients (all serum calcitonin levels were higher than the normal range) were high (> 21.0 pg/mL) in the present study. In addition, the FNA-Ct value and FNA-Ct/serum calcitonin ratio were higher in the hereditary MTC patients compared to the sporadic MTC patients. For this reason, we speculate that in the hereditary MTC patients, perhaps the C-cell hyperplasia in the areas surrounding non-MTC nodules was also punctured by the aspiration when FNAC was performed. Thus, in MTC patients the levels of serum calcitonin are high and the nodules for which the values of FNA-Ct are lower can be classified as negative, indicating the need for careful judgment regarding nodules with high FNA-Ct values, especially in hereditary MTC patients. In such cases, it may be effective to conduct an immunocytochemical analysis for calcitonin to identify MTC. However, the feasibility of this procedure is limited to cytologic samples with adequate cellularity, and MTCs may be not identified on inadequate or suboptimal cytologic specimens [20].

## Conclusions

In conclusion, this study is the first to identify the calcitonin cut-off value in fine-needle aspirate washout fluid for use with the currently available calcitonin assay system and an ECLIA. We propose that the calcitonin cut-off value in fine-needle aspirate washout fluid using a calcitonin assay system with an ECLIA is 21.0 pg/mL.

## Abbreviations

ATA: American Thyroid Association
AUC: Area under the curve
CEA: Carcinoembryonic antigen
ECLIA: Electrochemiluminescence immunoassay
FNAC: Fine-needle aspiration cytology
FNA-Ct: fine-needle aspirate washout fluid
MTC: Medullary thyroid carcinoma
PTC: Papillary thyroid carcinoma
RIA: Immunoradiometric assay
ROC: Receiver operating characteristic
